# A Comparison of Lipids and apoB in Asian Indians and Americans

**DOI:** 10.5334/gh.882

**Published:** 2021-01-20

**Authors:** Kavita Singh, George Thanassoulis, Line Dufresne, Albert Nguyen, Ruby Gupta, KM Venkat Narayan, Nikhil Tandon, Allan Sniderman, Dorairaj Prabhakaran

**Affiliations:** 1 Public Health Foundation of India, IN; 2 Centre for Chronic Disease Control, New Delhi, IN; 3 McGill University Health Centre, and Department of Medicine, McGill University, Montreal, CA; 4 Preventive and Genomic Cardiology, McGill University Health Centre and Research Institute, Montreal, Quebec, CA; 5 Rollins School of Public Health, Emory University, Atlanta, US; 6 Department of Endocrinology, All India Institute of Medical Sciences, New Delhi, IN; 7 London School of Hygiene and Tropical Medicine, UK

**Keywords:** dyslipidemia, apolipoprotein B, India, United States, atherosclerotic vascular disease

## Abstract

**Background and aims:**

Apolipoprotein B (apoB) integrates and extends the information from the conventional measures of atherogenic cholesterol and triglyceride. To illustrate how apoB could simplify and improve the management of dyslipoproteinemia, we compared conventional lipid markers and apoB in a sample of Americans and Asian Indians.

**Methods:**

Data from the US National Health and Nutrition Examination Survey (NHANES) (11,778 participants, 2009–2010, 2011–2012), and the Centre for Cardiometabolic Risk Reduction in South Asia (CARRS) cohort study in Delhi, India (4244 participants), 2011 were evaluated. We compared means and distributions of plasma lipids, and apo B using the Mann–Whitney U test and Fisher’s exact test. A p value of < 0.05 was considered significant.

**Results:**

The plasma lipid profile differed between Asian Indians and Americans. Plasma triglycerides were greater, but HDL-C lower in Asian Indians than in Americans. By contrast, total cholesterol, non-HDL-C, and LDL-C were all significantly higher in Americans than Asian Indians. However, apoB was significantly higher in Asian Indians than Americans. The LDL-C/apoB ratio and the non-HDL-C/apoB ratio were both significantly lower in Asian Indians than Americans.

**Conclusion:**

Whether Americans or Asian Indians are at higher risk from apoB lipoproteins cannot be determined based on their lipid levels because the information from lipids cannot be integrated. ApoB, however, integrates and extends the information from triglycerides and cholesterol. Replacing the conventional lipid panel with apoB for routine follow ups could simultaneously simplify and improve clinical care.

## Introduction

The conventional lipid panel can be complex and confusing. HDL cholesterol (HDL-C) is used to estimate risk, but not thereafter, even though it is always measured and reported. Of the four proatherogenic lipid markers- total cholesterol (TC), triglycerides (TG), low-density lipoprotein cholesterol (LDL-C), non-high-density lipoprotein cholesterol (non-HDL-C)- the major guidelines have designated LDL-C to be the principal one on which clinical decisions are based [1,2]. However, non-HDL-C appears to be a more accurate marker of cardiovascular risk than LDL-C and many authorities argue that TG is an independent risk factor for cardiovascular disease (CVD) and should be an independent target for therapy [3,4,5].

Therefore, why should TG and non-HDL-C not be taken into account as well as LDL-C and why should risk be calculated using TC rather than LDL-C or non-HDL-C? LDL-C is the gold standard on which guideline care is based, but there is no accepted gold standard to measure LDL-C in clinical care [2,6,7]. Should LDL-C be calculated and, if so, by which algorithm? Or should LDL-C be measured directly, and if so, by which method? Consensus reports of American and European Clinical Chemists as well the most recent ESC/EAS guidelines state that none are standardized and all are associated with considerable error, particularly at the low concentrations of LDL-C that are so common in the statin era [6,7]. No wonder the selection and care of subjects with or at high risk of cardiovascular remains imperfect in societies with developed medical systems and almost inconceivable in societies with much more limited resources. Or, is there is a simpler, better way forward.

The 2019 ESC/EAS Guidelines state there is [1,2]. Each atherogenic lipoprotein particle- chylomicron remnant particles, very low-density lipoprotein (VLDL) particles, intermediate-density lipoprotein (IDL) particles, LDL particles, and Lp(a) particles- contains one molecule of apoB: [8,9] apoB48 in the chylomicron remnant particles, apoB100 in the rest [5]. Plasma apoB, therefore, equals the total number of atherogenic particles [9]. LDL particles, almost always, make up the great majority of apoB particles [9]. However, except for intact chylomicron particles, which are too large to penetrate the arterial wall, all the other apoB particles are small enough to enter and to be trapped within it. The number of apoB particles within the lumen of the artery is the primary determinant of the number of apoB particles that will enter and be trapped within the arterial wall and it is the number of apoB particles with the cholesterol within them that are trapped within the arterial wall that initiates and drives the atherosclerotic process from beginning to end [9,10,11].

To illustrate how apoB could transform clinical care, we compared plasma lipids and apoB in Asian Indian and Americans by age-group and sex.

## Methods

### Study setting and populations

Data describing the American population were derived from the National Health and Nutrition Examination Survey (NHANES 2009-2010 & 2011-2012), which is designed to be representative of the United States non-institutionalized civilian population. Detailed methods used in NHANES are published elsewhere and are available for public access on the Internet [12]. The measurements of apoB were compliant with the WHO-IFCC apolipoprotein program as previously documented [13]. The coefficient of variation for TC was < 3%, TG < 4%, HDL-C < 4%, and apoB < 7%.

Data from India were derived from the Centre for Cardiometabolic Risk Reduction in South Asia (CARRS) study, which is a hybrid, cohort-modelled, cross-sectional multicentre surveillance study. The CARRS study was carried out in two cities, Delhi and Chennai, which are located in two culturally and geographically distinct Indian states and Karachi, Pakistan. The methods, participant recruitment, data collection in CARRS cohort study have been published elsewhere [14].

For this study, the data analysis was restricted to Delhi since apoB was not measured in Chennai or Karachi. Overall, 5365 participants from Delhi (response rate: 95%) were recruited during the baseline survey conducted between October 2010 and December 2011. Of the 5365 participants from Delhi, 4244 (80%) who had complete data on lipids fractions comprise the analytic group. In general, this group was representative of all participants from Delhi. Information on statin use was not available for either Asian Indians or Americans. CARRS study has received institutional ethics approval from the Public Health Foundation of India and study participants provided written informed consent prior their participation in this study.

### Measurements

Data on age, sex, and sociodemographic and lifestyle factors were obtained using a standardized questionnaire. A summary of all surveillance indicators, measures, methods and instruments used in the study has been published in detail [14]. Standard assay methods for assessment of diabetes (plasma glucose, haemoglobin A1c) and dyslipidemia (total cholesterol, LDL- cholesterol, HDL-cholesterol and triglycerides) were used. ApoB was measured using an immunoturbidimetric method (Roche Diagnostics, Switzerland). The intra-assay and inter-assay coefficient of variation were <3% and <5 % respectively. Two levels of internal controls were run with every batch of samples. The laboratory participated in External Quality Assurance program from RIQAS (Randox International Quality Assurance Scheme) for apoB.

### Statistical Analyses

To compare means and distributions of plasma lipids apoB between Asian Indians and Americans, Mann–Whitney U test and Fisher’s exact test were performed using R version 3.5.1. A p value of <0.05 was considered significant. The R package overlapping 1.5.0. was used to calculate the lipid biomarker density curve percent overlap.

## Results

Data on age, sex, plasma lipids and apoB were available in 11,788 Americans from the NHANES data base and 4244 Indians from Delhi (Table 1). The Americans were just over four years older than the Indians, but there were virtually an equal number of women and men in both groups. Nevertheless, the plasma lipid profile differed considerably between the two groups. Plasma TG was substantially greater (19%; p < 0.001), but HDL-C substantially lower (12%; P < 0.001) in Asian Indians than in Americans. By contrast, TC was 6%, non-HDL-C 3% and LDL-C 7% (all p < 0.001) greater in Americans than Asian Indians. Asian Indians, therefore, were relatively hypertriglyceridemic with low HDL-C compared to Americans whereas Americans were hypercholesterolemic compared to Asian Indians. On the other hand, the level of the atherogenic lipoproteins based on apoB was 7% (p < 0.001) higher in Asian Indians than Americans. Moreover, the LDL-C/apoB ratio and the non-HDL-C/apoB ratio were both significantly lower in Indians than Americans. Of interest, although the BMI and waist circumference were both lower in Indians compared to Americans, the waist to BMI ratio was greater in Indians than Americans pointing to greater abdominal obesity in Indians than Americans.

**Table 1:** Characteristics of the study populations from India and the United States.

	US (N = 11778) Median [IQR] or n (%)	India (N = 4244) Median [IQR] or n (%)	p-value

**Age**	49 [34–64]	44 [35–54]	< 0.001
**Gender (Female)**	6032 (51.2)	2142 (50.5)	0.410
**ApoB (mg/dL)**	89 [73–107]	95 [79–112]	< 0.001
**TG (mg/dL)**	106 [76–152]	126 [94–175]	< 0.001
**TC (mg/dL)**	192 [165–220]	181 [153–206]	< 0.001
**Non-HDL-C (mg/dL)**	138 [112–167]	134 [108–160]	< 0.001
**LDL-C (mg/dL)**	112 [90–137]	105 [83–127]	< 0.001
**LDL-C / ApoB ratio**	1.3 [1.2–1.4]	1.1 [1.0–1.2]	< 0.001
**Non-HDL-C / ApoB ratio**	1.5 [1.4–1.6]	1.4 [1.3–1.5]	< 0.001
**HDL-C (mg/dL)**	50 [41–61]	44 [37–52]	< 0.001
**BMI (kg/m^2^)**	28 [24–32]	25 [22–29]	< 0.001
**Waist (cm)**	97 [87–108]	88 [80–97]	< 0.001
**Waist / BMI ratio**	3.5 [3.3–3.7]	3.5 [3.2–3.8]	0.035

ApoB = apolipoprotein B mg/dL, TG = triglycerides mg/dL, TC = total cholesterol mg/dL, LDL-C = low-density lipoprotein cholesterol mg/dL, HDL-C = high-density lipoprotein cholesterol mg/dL; BMI = body mass index; Kg/m^2^ = kilogram per meter square, cm = centimeter; mg/dL = milligram per decilitre; IQR = interquartile range; US = United States.

**Figure 1:**
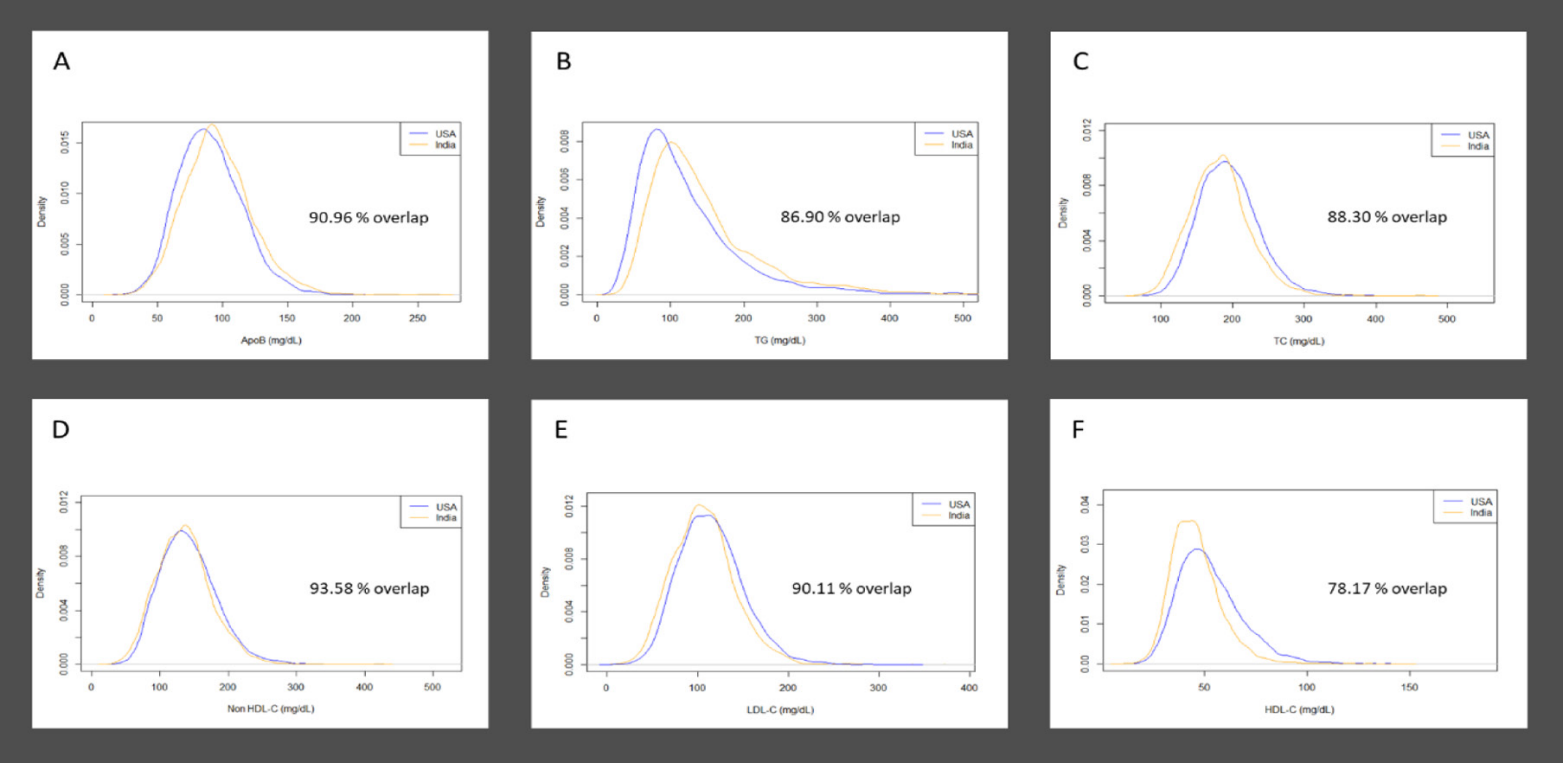
Density Plots for the lipid biomarkers. A: ApoB = apolipoprotein B mg/dL; B: TG = triglycerides mg/dL; C: TC = total cholesterol mg/dL; D: Non-HDL-C = total cholesterol mg/dL – high-density lipoprotein cholesterol mg/dL; E: LDL-C = low-density lipoprotein cholesterol mg/dL; F: HDL-C = high-density lipoprotein cholesterol mg/dL; USA = United States of America.

The differences in levels of lipids and apoB are demonstrated in more detail in Figure 1. The most obvious differences are seen in the levels of triglyceride and HDL-C with the distribution of triglyceride levels shifted to the right, i.e. higher, while the distribution of HDL-C levels is shifted to the left, i.e. lower, in Asian Indians compared to Americans. By contrast, the distributions of the levels of TC, LDL-C, and non-HDL-C were higher in Americans compared to Asian Indians while the distribution of apoB was higher in Asian Indians than Americans. As illustrated in Figure 1, while the degree of overlap for any marker is considerable, there is less overlap for TG and especially HDL-C between the two groups while the overlap for apoB and the cholesterol markers is much greater.

Results from the two populations are also compared by quintiles in Table 2. The greatest differences between the two populations are present for TG and HDL-C. TG are substantially higher in Asian Indians than Americans whereas HDL C is substantially lower in Asian Indians than Americans. By contrast, TC, non-HDL-C and LDL-C are all consistently higher in Americans than Asian Indians while apoB is consistently higher in Asian Indians than Americans.

**Table 2:** Quantiles and percentiles of lipids and apoB in Indians and Americans.

	ApoB		TG		TC		Non-HDL-C		LDL-C		HDL-C	

	India	US	India	US	India	US	India	US	India	US	India	US

**Min**	22	15	30	26	70	59	30	23	11.0	9	10	14
**25%**	79	73	94	76	153	165	108	112	83.0	90	37	41
**50%**	95	89	126	106	181	192	134	138	105.0	112	44	50
**Mean**	97	91	151	130	182	194	136	142	107	115	46	53
**75%**	112	107	175	152	206	220	160	167	127.0	137	52	61
**80%**	117	111	196	168	213	227	167	175	133	143	54	64
**90%**	130	122	250	219	234	247	189	195	151	161	61	73
**Max**	264.4	260	2112	2742	467	528	421	504	373	331	147	179

ApoB = apolipoprotein B mg/dL, TG = triglycerides mg/dL, TC = total cholesterol mg/dL, LDL-C = low-density lipoprotein cholesterol mg/dL, HDL-C = high-density lipoprotein cholesterol mg/dL; US = United States.

Results by age and sex are presented in Table 3. At virtually every age, in both males and females, with only a handful of exceptions, such as LDL-C and non-HDL-C in males at the two oldest ages, the differences observed in the overall results are evident in each of the specific comparisons. When adjusted by age and sex, the differences for apoB between Asian Indians and Americans remained highly significant (p < 0.001) whereas the differences for non-HDL-C were no longer significant (p NS). The overall results, therefore, are the product of a consistent pattern of differences between Asian Indians and Americans at all ages and in both sexes.

**Table 3:** Lipids and apoB by age and sex.

Males		20<= Age <=29	30<= Age <=39	40<= Age <=49	50<= Age <=59	60<= Age <=69	Age >=70

N	US	138	162	200	156	134	271
	New Delhi	230	445	585	404	237	118
TC	US	166 [147, 194]	188 [169, 212]	198 [174, 221]	194 [170, 218]	186 [156, 212]	171 [148, 204]
	New Delhi	157 [132, 183]	179 [156, 201]	181 [154, 207]	187 [163, 210]	184 [158, 207]	175 [145, 203]
TG	US	107 [70, 137]	120 [83, 151]	123 [88, 176]	121 [90, 167]	102 [78, 138]	107 [81, 147]
	New Delhi	110 [82, 156]	137 [98, 193]	138 [106, 201]	136 [106, 183]	132 [98, 175]	118 [92, 150]
HDL-C	US	48 [40, 56]	45 [39, 52]	46 [39, 53]	46 [39, 54]	49 [41, 62]	47 [40, 57]
	New Delhi	41 [35, 49]	41 [35, 49]	41 [35, 48]	43 [36, 51]	43 [35, 53]	43 [38, 50]
ApoB	US	74 [61, 91]	91 [78, 108]	96 [82, 111]	93 [78, 109]	87 [74, 103]	83 [69, 98]
	New Delhi	81 [66, 101]	96 [81, 113]	97 [84, 116]	100 [85, 117]	97 [85, 116]	92 [77, 111]
LDL-C	US	97 [77, 120]	118 [100, 134]	123 [101, 141]	117 [95, 142]	110 [86, 131]	97 [77, 123]
	New Delhi	85 [67, 110]	103 [80, 125]	105 [84, 128]	111 [89, 134]	110 [85, 130]	103 [80, 125]
Non HDLC	US	117 [98, 148]	145 [124, 163]	151 [130, 172]	146 [119, 167]	132 [105, 154]	122 [100, 151]
	New Delhi	113 [87, 141]	136 [110, 159]	137 [114, 161]	141 [115, 164]	139 [111, 160]	129 [102, 154]
LDL-C/ApoB ratio	US	1.31 [1.2, 1.4]	1.31 [1.2, 1.4]	1.29 [1.2, 1.4]	1.27 [1.2, 1.4]	1.25 [1.1, 1.4]	1.18 [1.1, 1.3]
	New Delhi	1.08 [1.0, 1.2]	1.08 [1.0, 1.2]	1.09 [1.0, 1.2]	1.11 [1.0, 1.2]	1.10 [1.0, 1.2]	1.12 [1.0, 1.2]
Non HDL-C/ApoB ratio	US	1.60 [1.5, 1.67]	1.58 [1.5, 1.67]	1.58 [1.5, 1.7]	1.55 [1.5, 1.6]	1.48 [1.4, 1.6]	1.48 [1.4, 1.6]
	New Delhi	1.35 [1.3, 1.5]	1.39 [1.3, 1.5]	1.40 [1.3, 1.5]	1.39 [1.3, 1.5]	1.38 [1.3, 1.5]	1.37 [1.3, 1.5]
BMI	US	26 [21, 30]	28 [25, 32]	29 [25, 32]	28 [25, 33]	28 [25, 33]	28 [25, 31]
	New Delhi	22 [19, 25]	24 [21, 27]	25 [22, 28]	25 [22, 29]	25 [22, 29]	23 [21, 26]
Waist	US	88 [79, 102]	98 [91, 108]	102 [93, 112]	105 [95, 117]	107 [96, 117]	105 [98, 113]
	New Delhi	80 [73, 87]	87 [79, 95]	92 [84, 99]	93 [85, 100]	94 [84, 102]	90 [83, 99]
Waist/BMI ratio	US	3.50 [3.4, 3.8]	3.53 [3.4, 3.7]	3.59 [3.4, 3.8]	3.66 [3.5, 3.8]	3.68 [3.5, 3.9]	3.78 [3.6, 4.0]
	New Delhi	3.61 [3.4, 3.8]	3.63 [3.4, 3.8]	3.65 [3.5, 3.9]	3.69 [3.5, 4.0]	3.71 [3.5, 4.0]	3.88 [3.7, 4.1]
**Females**		**20<= Age <=29**	**30<= Age <=39**	**40<= Age <=49**	**50<= Age <=59**	**60<= Age <=69**	**Age >=70**

N	US	165	197	179	164	144	294
	New Delhi	251	555	630	357	247	83
TC	US	173 [152, 195]	185 [163, 213]	195 [174, 222]	217 [185, 242]	207 [188, 232]	199 [175, 230]
	New Delhi	150 [126, 171]	166 [144, 194]	185 [162, 208]	196 [175, 217]	195 [170, 219]	196 [169, 222]
TG	US	92 [64, 121]	89 [65, 131]	92 [66, 145]	112 [78, 164]	108 [78, 158]	122 [92, 159]
	New Delhi	87 [63, 120]	109 [83, 144]	124 [94, 165]	135 [100, 182]	129 [102, 179]	129 [99, 160]
HDL-C	US	53 [44, 65]	55 [48, 68]	58 [46, 69]	59 [48, 68]	58 [47, 72]	58 [48, 69]
	New Delhi	46 [40, 54]	46 [39, 53]	46 [39, 54]	48 [41, 56]	47 [41, 57]	52 [46, 60]
ApoB	US	77 [64, 88]	82 [67, 98]	86 [72, 106]	94 [79, 115]	92 [78, 109]	91 [78, 108]
	New Delhi	77 [64, 88]	86 [72, 102]	97 [83, 111]	104 [88, 117]	103 [89, 118]	97 [81, 116]
LDL-C	US	97 [81, 121]	107 [87, 131]	115 [95, 140]	126 [100, 155]	119 [100, 149]	112 [90, 139]
	New Delhi	84 [66, 100]	95 [78, 118]	109 [90, 130]	119 [100, 137]	115 [95, 136]	115 [93, 136]
Non HDLC	US	119 [100, 141]	128 [106, 152]	137 [112, 166]	150 [122, 186]	143 [127, 171]	139 [116, 169]
	New Delhi	103 [83, 123]	118 [98, 144]	137 [113, 158]	149 [124, 169]	144 [121, 169]	140 [117, 165]
LDL-C/ApoB ratio	US	1.30 [1.2, 1.4]	1.34 [1.3, 1.4]	1.35 [1.2, 1.4]	1.34 [1.2, 1.4]	1.32 [1.2, 1.4]	1.24 [1.1, 1.4]
	New Delhi	1.10 [1.0, 1.2]	1.12 [1.0, 1.2]	1.13 [1.0, 1.2]	1.14 [1.1, 1.2]	1.14 [1.1, 1.2]	1.15 [1.1, 1.2]
Non HDL-C/ApoB ratio	US	1.54 [1.5, 1.6]	1.58 [1.5, 1.7]	1.56 [1.5, 1.7]	1.59 [1.5, 1.7]	1.58 [1.5, 1.7]	1.53 [1.4, 1.6]
	New Delhi	1.35 [1.3, 1.4]	1.38 [1.3, 1.5]	1.40 [1.3, 1.5]	1.41 [1.3, 1.5]	1.40 [1.3, 1.5]	1.42 [1.3, 1.5]
BMI	US	26 [22, 30]	27 [23, 32]	27 [22, 34]	28 [24, 33]	29 [25, 34]	27 [24, 32]
	New Delhi	22 [19, 26]	25 [22, 29]	27 [24, 31]	28 [25, 33]	27 [23, 31]	27 [23, 30]
Waist	US	89 [79, 99]	91 [83, 106]	94 [82, 107]	95 [88, 106]	100 [88, 111]	97 [88, 107]
	New Delhi	76 [67, 84]	84 [75, 91]	89 [81, 96]	91 [84, 99]	91 [83, 99]	90 [84, 97]
Waist / BMI ratio	US	3.41 [3.2, 3.7]	3.43 [3.2, 3.6]	3.44 [3.2, 3.7]	3.46 [3.2, 3.7]	3.40 [3.2, 3.6]	3.54 [3.3, 3.8]
	New Delhi	3.37 [3.1, 3.6]	3.26 [3.1, 3.6]	3.24 [3.0, 3.5]	3.22 [3.0, 3.5]	3.34 [3.1, 3.6]	3.43 [3.2, 3.7]

N = total sample; ApoB = apolipoprotein B mg/dL, TG = triglycerides mg/dL, TC = total cholesterol mg/dL, LDL-C = low-density lipoprotein cholesterol mg/dL, HDL-C = high-density lipoprotein cholesterol mg/dL; BMI = body mass index; Kg/m^2^ = kilogram per meter square, cm = centimeter; mg/dL = milligram per decilitre; IQR = interquartile range; US = United States.

## Discussion

The conventional lipid panel is complex and can be confusing and contradictory. In this study, all the cholesterol markers are higher in the Americans whereas triglycerides are higher and HDL-C lower in the Asian Indians. Which population is at higher risk due to the apoB lipoproteins? Because non-HDL-C includes the cholesterol in VLDL particles, non-HDL-C is thought to compensate for the fact that LDL-C underestimates LDL particle number in patients with hypertriglyceridemia [9]. But the mass of cholesterol within LDL particles is the primary determinant of non-HDL-C [9], which explains why non-HDL-C is higher in Americans, notwithstanding their triglycerides are lower than Asian Indians. By contrast, apoB was higher in Asian Indians than Americans and apoB, as we discuss later to contextualize our study findings, is a more accurate index of risk than non-HDL-C. Interestingly, cardiovascular disease may be even more prevalent in Indians than in Americans [15].

The 2019 European Society of Cardiology/European Atherosclerosis (ESC/EAS) Guidelines concluded that apoB is superior to LDL-C and non-HDL-C as a marker of cardiovascular risk and the adequacy of LDL lowering therapy [2]. This judgement was based on a wealth of evidence including a meta-analysis of prospective observational studies as well as multiple meta-analyses of randomized clinical trials [3,16,17,18,19], multiple discordance analyses [20,21,22,23,24,25,26,27,28], plus Mendelian randomization-based analyses of prospective observational studies and randomized clinical trials [29,30]. The positive judgement of the 2019 ESC/EAS Guidelines in favour of apoB marks a turning point in the debate as to which is the most accurate marker of cardiovascular risk-LDL-C, non-HDL-C or apoB. The residual reservations of ESC/EAS to apoB replacing LDL-C were lack of familiarity of physicians with this new marker and its lack of availability at the moment, for routine clinical care.

In this regard, the 2019 ESC/EAS Guidelines stated that apoB could be measured accurately and inexpensively on non fasting samples using standardized methods that could be easily available in almost all clinical chemistry laboratories [2]. By contrast, they noted substantial challenges in the estimation of LDL-C and non-HDL-C, particularly at the low levels that are common in this era of potent statin plus perhaps ezetimibe and possibly PCSK9 therapy [2,6,7]. Detailed review previously by the American College of Clinical Chemistry and, more recently, by the European Atherosclerosis Society/European Federation of Laboratory Medicine confirm and extend these judgements [6,7]. Superior laboratory performance is, on its own, an unanswerable argument for the widespread clinical introduction of apoB.

But there is a new and powerful argument in favour of apoB. Rather than merely adding apoB to a conventional lipid panel, apoB could replace a conventional lipid panel for routine visits, simultaneously improving and simplifying clinical care. With the exception of type III hyperlipoproteinemia, apoB48 particles contribute minimally to total apoB, which is determined by the sum of VLDL and LDL particles [9]. Trapping of apoB particles within the arterial wall initiates and promotes the atherosclerotic process from beginning to end [10]. Larger cholesterol-enriched particles will deposit more cholesterol than smaller cholesterol-depleted particles but smaller cholesterol-depleted apoB particles are more likely to enter and be trapped within the arterial wall than larger cholesterol-enriched apoB particles [29,31,32]. Accordingly, LDL particles, regardless of their cholesterol content, appear to be equally atherogenic.

Evidence of the equality of VLDL and LDL particles as promoters of atherogenesis comes from Ference and colleagues, who utilized Mendelian randomization to demonstrate that the benefit of lowering either triglycerides or LDL-C was the same per 10 mg/dl lower apoB [30]. This means that the atherogenic risk of one VLDL particle is the same as the atherogenic risk of one LDL particle. Ference et al. also showed that the genetic equivalents of multiple therapeutic agents, which have been shown to reduce triglyceride by a variety of different mechanisms, all produce clinical benefit in proportion to the lowering of apoB, not the lowering of triglyceride or LDL-C [30]. Their findings confirm those reported previously in a metabolic profiling study by Wurtz et al. [32], which demonstrated that the relative risk associated with multiple VLDL and LDL subfractions did not differ substantially. Furthermore, Ohukainen and colleagues used artificial intelligence to create a self-organizing map, which resulted in four lipid phenotypes. However, apoB alone produced better resolution of risk with a striking dose-response relation to cardiovascular risk [33]. This equivalence of the atherogenic risk associated with VLDL and LDL would explain why statins have so consistently produced unequivocal evidence clinical benefit whereas fibrates have not [9]. But the explanatory power of apoB appears to go even further. The multivariable Mendelian randomization analysis by Richardson and his colleagues [34], a first of its kind, demonstrated that once apoB was taken into account, neither triglycerides, nor LDL-C, nor HDL-C retained significant independent predictive relationships to atherosclerotic cardiovascular risk. This is the first analysis to demonstrate that apoB encapsulates the risk from both the apoB and the apoA lipoprotein particles. Finally, the recent Mendelian randomization analysis by Zuber et al. [35], using a different analytic and statistical approach than Richardson, an approach which encompassed more than 30 lipid variables and metabolites, whose individual and collective significance were assessed by Bayesian methods, confirmed that apoB is the key lipid determinant of cardiovascular risk because it incorporates the risk associated with all the atherogenic components within VLDL and LDL particles including triglycerides and cholesterol.

There are, nevertheless, two exceptions to the rule that apoB captures the full atherogenic potential of the apoB lipoproteins: Lp(a) and the abnormally cholesterol-rich remnant particles in type III hyperlipoproteinemia. Type III hyperlipoproteinemia is an uncommon disorder, which cannot be diagnosed based on a conventional lipid panel but can be accurately recognized based on total cholesterol, triglyceride and apoB [36]. Lp(a) does increase the risk of atherosclerosis beyond that captured by apoB but this may only matter in those whose LDL-C or apoB are also elevated [37,38]. Unfortunately, in contrast to apoB, standardized assays for Lp(a) are not yet available.

Our study has important limitations. Most important is that the lipid and apoB results are not directly related to clinical outcomes within these study groups. However, the multiple lines of evidence we cite that demonstrate apoB is superior to LDL-C and non-HDL-C include studies such as INTERHEART and INTERSTROKE [24,39,40], which include subjects from all regions of the world. The NHANES sample is selected to be representative of the non-military, non-institutionalized population of the United States. The participants from India, however, represent an urban segment of a larger CARRS cohort study and cannot be extrapolated to all Asian Indians. All laboratory measurements were performed using standardized methods. The results, therefore, should be comparable. However, it is not possible to arrange for cross-testing of methods to demonstrate this directly. Nevertheless, all meta-analyses comparing apoB with LDL-C or non-HDL-C have made a similar presumption.

In conclusion, this study illustrates how apoB can integrate different patterns of abnormal plasma lipid levels so that one becomes comparable to the other. The conventional lipid panel is complex with multiple markers, whose clinical significance, one versus the other, cannot be meaningfully integrated. The more complex a diagnostic and therapeutic system is for the care-provider and the patient understand and apply, the less successful care will be. Conversely, the simpler a diagnostic and therapeutic system is for the care-provider and the patient to understand and apply, the more successful care will be ApoB can be measured accurately and inexpensively on non-fasting samples using equipment that is already available in almost all laboratories that now measure or calculate LDL-C [2,6,7]. Based on apoB, less expensive, simpler, more effective systems to deliver care to prevent and treat cardiovascular disease could be implemented in developing as well as developed countries.

## Data Availability Statement

Dr Kavita Singh, Dr. Sniderman and Dr. Prabhakaran have access to the study dataset and will be happy to share with other researchers upon request sent to the corresponding authors: Dr. Prabhakaran and Dr. Sniderman.

## Figures and Tables

**Table:** 

ApoB	Apolipoprotein B
BMI	Body mass index
CARRS	Centre for Cardiometabolic Risk Reduction in South Asia
CVD	Cardiovascular disease
HDL-C	High-density lipoprotein cholesterol
IDL	Intermediate density lipoprotein
LDL-C	Low-density lipoprotein cholesterol
NHANES	National Health and Nutrition Examination Survey
TC	Total cholesterol
TG	Triglycerides
US	United States
VLDL	Very low-density lipoprotein
